# Dating early animal evolution using phylogenomic data

**DOI:** 10.1038/s41598-017-03791-w

**Published:** 2017-06-15

**Authors:** Martin Dohrmann, Gert Wörheide

**Affiliations:** 1 Ludwig-Maximilians-Universität München, Department of Earth & Environmental Sciences, Palaeontology & Geobiology, Richard-Wagner-Str. 10, 80333 Munich, Germany; 2 GeoBio-CenterLMU, Ludwig-Maximilians-Universität München, Richard-Wagner Str. 10, 80333 Munich, Germany; 3 SNSB – Bavarian State Collections of Palaeontology and Geology, Richard-Wagner Str. 10, 80333 Munich, Germany

## Abstract

Information about the geological timeframe during which animals radiated into their major subclades is crucial to understanding early animal ecology and evolution. Unfortunately, the pre-Cambrian fossil record is sparse and its interpretation controversial. Relaxed molecular-clock methods provide an alternative means of estimating the timing of cladogenesis deep in the metazoan tree of life. So far, thorough molecular clock studies focusing specifically on Metazoa as a whole have been based on relatively small datasets or incomplete representation of the main non-bilaterian lineages (such as sponges and ctenophores), which are fundamental for understanding early metazoan evolution. Here, we use a previously published phylogenomic dataset that includes a fair sampling of all relevant groups to estimate the timing of early animal evolution with Bayesian relaxed-clock methods. According to our results, all non-bilaterian phyla, as well as total-group Bilateria, evolved in an ancient radiation during a geologically relatively short time span, before the onset of long-term global glaciations (“Snowball Earth”; ~720–635 Ma). Importantly, this result appears robust to alterations of a number of important analytical variables, such as models of among-lineage rate variation and sets of fossil calibrations used.

## Introduction

Our understanding of the origin and evolution of animals (Metazoa) and their major subgroups would be greatly enlightened by better knowledge about the timing of diversification early in their history. Metazoa comprises five main lineages (see Dohrmann & Wörheide^1^ for a review of their phylogenetic relationships): the phyla Porifera (sponges), Placozoa (*Trichoplax*), Ctenophora (comb jellies), Cnidaria (corals, jellyfish, and their kin), and Bilateria, the mega-diverse group containing all the remaining 30 or so phyla. Most animal phyla appear in the fossil record in a relatively short period during the Cambrian (541–485 million years ago [Ma]), the so-called “Cambrian explosion”^2–4^. However, these Cambrian fossils already exhibit complex morphologies, supporting the idea that animals must have evolved some time during the Proterozoic (2500–541 Ma). Indeed, it is now widely agreed that animals existed at least during the later stages of the preceding Ediacaran period (635–541 Ma), as evidenced by trace fossils and some body fossils with likely metazoan affinities^5–7^. Unfortunately, the older fossil record is relatively scarce with respect to possible animals, and most findings are being controversially discussed^8^, which prevents reliable inferences about the origination times of the five main lineages. Therefore, molecular palaeobiological approaches^9, 10^ might aid in deciphering early animal evolution by means of divergence time estimation from genomic data of extant species (molecular clock studies).

Although molecular clock studies have generally supported a deep pre-Ediacaran origin of animals and many of its major subgroups^11–14^, estimates of the precise timing vary widely^8, 15^. Moreover, the majority of studies have focused either on estimating the timescale of eukaryote evolution and thus including only few animals as representatives of just one lineage within the Opisthokonta supergroup^13, 16^, or severely undersampled the full diversity of Metazoa because they were mainly interested in the evolution of Bilateria^14, 17^. However, reliably estimating the timing of early metazoan evolution requires that all non-bilaterian lineages be adequately represented.

In a highly cited molecular clock study, Erwin *et al*.^12^ included a variety of non-bilaterians – 20 sponges, *Trichoplax*, and six cnidarians. However, they did not include Ctenophora and only sampled three of the four classes of Porifera (Hexactinellida was excluded). Furthermore, the dataset they used for dating their trees is comparatively small, being composed of sequence data from seven proteins only (~2000 amino acid positions). Thus, the results of Erwin *et al*.^12^ remain to be tested with a more complete taxon sampling and phylogenomic-scale data. Philippe *et al*.^18^ used a 128-protein phylogenomic dataset (30,257 amino acid positions) to reconstruct animal phylogeny. This dataset includes all non-bilaterian phyla and classes, a selected sample of representatives from all three bilaterian supergroups (Deuterostomia, Ecdysozoa, Lophotrochozoa), as well as a suite of non-metazoan opisthokonts as outgroups. Supporting a well-resolved phylogeny, this dataset appears well suited for investigating the timing of deep metazoan phylogeny. However, in-depth molecular clock studies utilizing this dataset have thus far been lacking. Here, we present molecular clock analyses of the Philippe *et al*.^18^ dataset under a variety of analytical conditions. Our results confirm previous molecular-clock estimates of an early-mid Neoproterozoic (Tonian; 1000–720 Ma) origin of crown-group Metazoa, before the onset of long-lasting global glaciations, the Sturtian and Marinoan “Snowball Earths” of the Cryogenian (720–635 Ma^19–21^). In contrast to previous studies, however, our results suggest that not only crown-group Metazoa, but all non-bilaterian phyla (and at least stem-lineages of all classes), as well as total-group Bilateria, originated before the Sturtian, probably within a geologically relatively short time span. Importantly, this main conclusion is robust to a number of major assumptions that can drastically influence the outcome of molecular clock studies.

## Methods

We conducted a range of relaxed molecular-clock analyses using the Bayesian Markov Chain Monte Carlo (MCMC) implementation PhyloBayes^22^. In order to assess the robustness of our age estimates to a number of prior assumptions, we ran analyses under (1) an autocorrelated and an uncorrelated relaxed molecular-clock model – the former assuming that the rate of molecular evolution of a lineage is correlated with the rate of its mother lineage, the latter allowing completely independent rates from lineage to lineage; (2) three different sets of fossil calibrations for internal nodes – one aiming at a maximum breadth of calibrations (*Set A*), another one excluding some potentially controversial fossils (*Set B*), and a third one adopted from Erwin *et al*.^12^ (*Set C*); (3) different prior assumptions about the age of the root of the phylogeny (=origin of crown-group Opisthokonta) – 800, 1000, and 1360 Ma; and 4) three different alternative assumptions about the phylogenetic placement of Ctenophora (sister to Cnidaria, sister to the remaining Metazoa, and sister to Placozoa + Cnidaria + Bilateria), which is currently a matter of debate^18, 23–26^. More detailed descriptions of these, as well as some additional, analyses are given in the Supplementary Material.

## Results

### Influence of molecular-clock model

**Figure 1:**
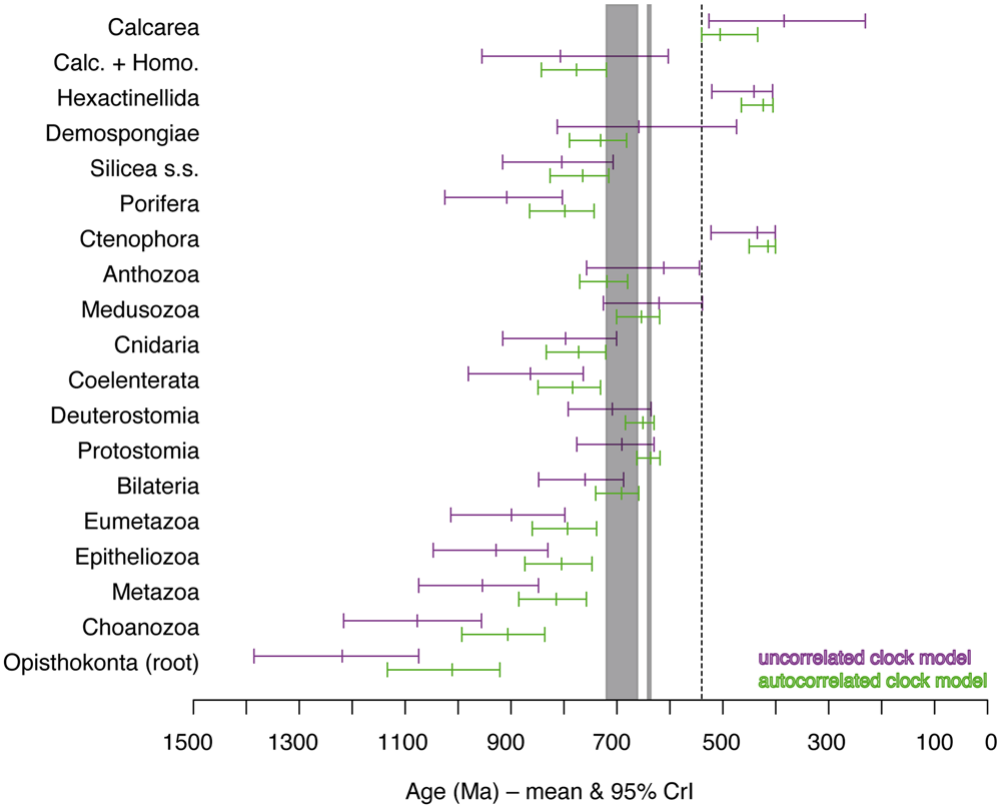
Age estimates for major animal groups obtained under different molecular clock models. Mean and 95% credibility intervals (CrIs) of age estimates for select nodes obtained with *Calibration set A* and the 1000 Ma root age prior (see text) under the autocorrelated “ln” (lower bars) and uncorrelated “ugam” (upper bars) relaxed clock models. Taxon names refer to crown groups. “Calc. + Homo.” = Calcarea + Homoscleromorpha clade. Ma = million years before present. Dotted line indicates Precambrian/Cambrian boundary. Gray areas indicate Sturtian (left) and Marinoan (right) glaciations^21^.

Mean age estimates for the nodes of greatest interest were generally older, sometimes considerably so, under the uncorrelated model (Fig. 1). The only exceptions were the crown-groups of Medusozoa (jellyfish and their kin), Anthozoa (corals, anemones etc.), Demospongiae (common sponges), and Calcarea (calcareous sponges), but the 95% credibility intervals (CrIs) obtained under the autocorrelated model for these nodes fell completely or almost completely within the CrIs obtained under the uncorrelated model. In general, the uncorrelated model yielded substantially wider CrIs, sometimes with ranges of hundreds of millions of years (e.g., Calcarea, Demospongiae). Under both models however, Metazoa and its deepest subclades – Porifera, Epitheliozoa (Placozoa + Eumetazoa), Eumetazoa (Coelenterata [=Cnidaria + Ctenophora] + Bilateria), and total-group Bilateria – as well as the crown-groups of Coelenterata, Cnidaria, Porifera, Silicea (siliceous sponges), and Calcarea + homoscleromorph sponges were estimated to have arisen before the Sturtian glaciation (the first of the Neoproterozoic Snowball Earth episodes). Given that the autocorrelated model had a better fit according to preliminary model selection analyses (see Supplementary Material) and yielded younger – i.e., in better agreement with the fossil record – and more precise age estimates for most nodes, we did not consider the uncorrelated model further.

### Influence of different fossil calibration sets

**Figure 2:**
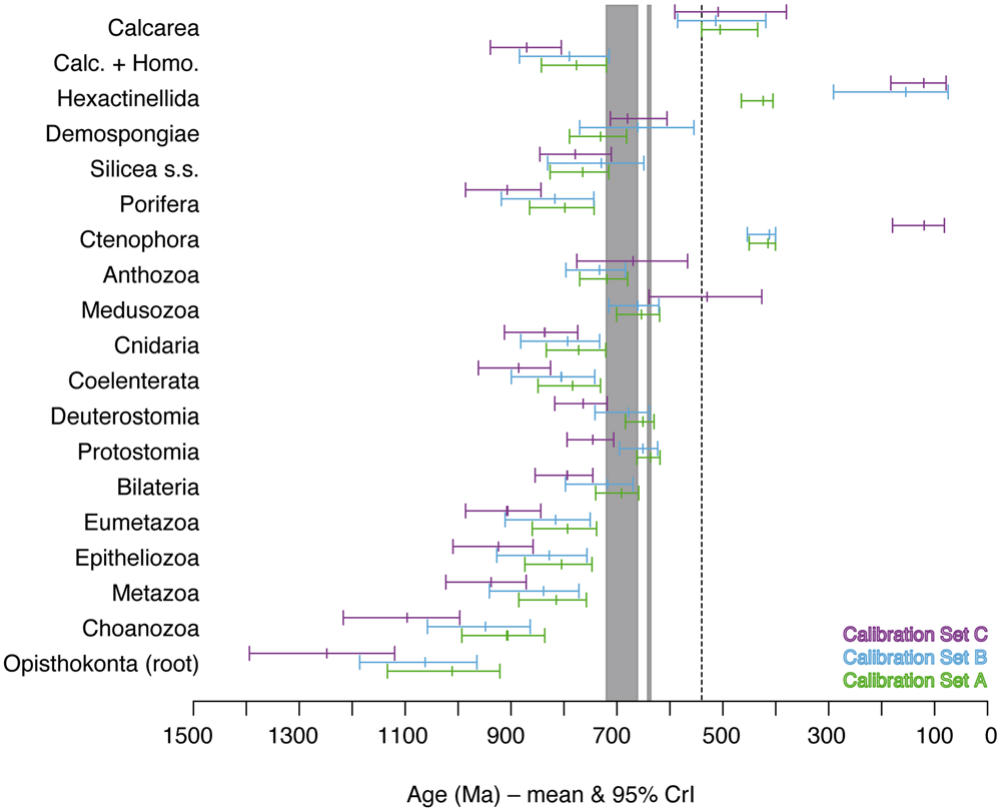
Age estimates for major animal groups obtained under different fossil calibration sets. Mean and 95% CrIs of age estimates for select nodes obtained under the autocorrelated “ln” relaxed clock model and the 1000 Ma root age prior using different fossil calibration sets (see text) – *A* (lower bars), *B* (middle bars), *C* (upper bars). Taxon names refer to crown groups. “Calc. + Homo.” = Calcarea + Homoscleromorpha clade. Dotted line indicates Precambrian/Cambrian boundary. Gray areas indicate Sturtian (left) and Marinoan (right) glaciations^21^.

For the deepest nodes (Opisthokonta to Eumetazoa), all three calibration sets resulted in pre-Sturtian age estimates, with *C* yielding the oldest, *A* the youngest, and *B* intermediate estimates (Fig. 2). The same pattern was also obtained for the crown groups of Coelenterata, Cnidaria, Porifera, and Calcarea + Homoscleromorpha. For crown Bilateria, Protostomia, and Deuterostomia, the ranking was the same, but estimates fell within the Sturtian, Marinoan, or interglacial intervals under *A* and *B*. Under *A* and *B*, CrIs for crown Medusozoa spanned the general glaciation interval, with means falling within or close to the interglacial period. In contrast, *C* yielded a very wide CrI with almost no overlap with those of *A* and *B*, with the mean much younger, falling in the earliest Cambrian. The pattern for crown Anthozoa was similar, but all estimates were older and consistent with a Sturtian origin. Mean estimates for crown Silicea were all pre-Sturtian and CrIs broadly overlapped. Estimates for crown Demospongiae were all consistent with a glacial origin, although the mean estimates were pre-Sturtian under *A*, Sturtian under *C*, and end-Sturtian under *B*. Only three of the nodes of greatest interest were estimated to be Phanerozoic: crown-group Calcarea, Hexactinellida (glass sponges), and Ctenophora. Mean estimates for Calcarea were mid-Cambrian under all three calibration sets, with CrIs narrowest for *A* and widest for *C*. The mean estimate for Hexactinellida (actually Hexasterophora: no members of the second subclass, Amphidiscophora, are included) was late Silurian under *A*, whereas under *B* and *C*, which did not constrain this node, much younger and certainly incorrect^27^ estimates (Upper Jurassic and Lower Cretaceous, respectively) were obtained. The situation for crown Ctenophora was very similar: Lower Devonian with the minimum constraint enforced (*A* and *B*), and Lower Cretaceous without the constraint (*C*). Overall, set *A* gave the most plausible and precise results, especially regarding the deepest nodes, which were excessively old under sets *B* and *C*. Furthermore, the calibration set of Erwin *et al*.^12^, which we used for set *C* has been heavily criticized^28^, so results obtained under set *C* have to be interpreted with caution. Therefore, we focus on results obtained with calibration set *A* in the remainder of the paper.

### Influence of root age prior

**Figure 3:**
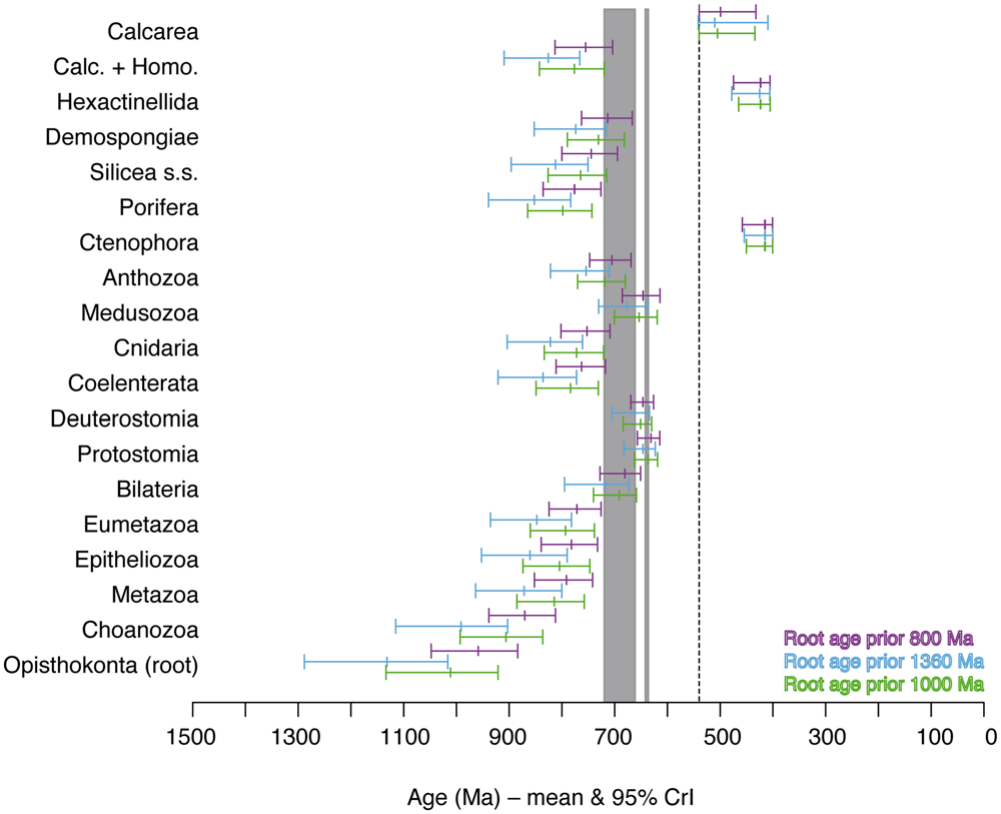
Age estimates for major animal groups obtained under different assumptions about the age of crown-group Opisthokonta. Mean and 95% CrIs of age estimates for select nodes obtained with *Calibration set A* (see text) under the autocorrelated “ln” relaxed clock model using different root age priors – 1000 ± 100 Ma (lower bars), 1360 ± 100 Ma (middle bars), 800 ± 100 Ma (upper bars). Taxon names refer to crown groups. “Calc. + Homo.” = Calcarea + Homoscleromorpha clade. Dotted line indicates Precambrian/Cambrian boundary. Gray areas indicate Sturtian (left) and Marinoan (right) glaciations^21^.

Not surprisingly, for almost all nodes of major interest, age estimates were younger than those obtained under the 1000 Ma root age prior when 800 Ma was assumed for the root, and older when 1360 Ma was assumed (Fig. 3); the only exceptions were crown-group Hexactinellida and Ctenophora, which had almost identical age estimates under the three different root age priors. However, the differences were not as big as we had anticipated. Particularly, the in our opinion unrealistically young root age prior of 800 Ma did not result in a major shift of age estimates towards after the Marinoan glaciation. In fact, the inference that Metazoa and most of its major subgroups originated prior to (or, regarding some crown clades, during) the Cryogenian Snowball Earth periods was not affected by assumptions about the age of crown-group Opisthokonta. In the following, we focus on the results obtained under the 1000 Ma root age prior, for better comparability with the results of Erwin *et al*.^12^ (also note that the 1360 Ma root age prior^13^ effectively represents a secondary calibration constraint, which renders the results obtained under this prior somewhat dubious^29, 30^).

### Influence of tree topology

**Figure 4:**
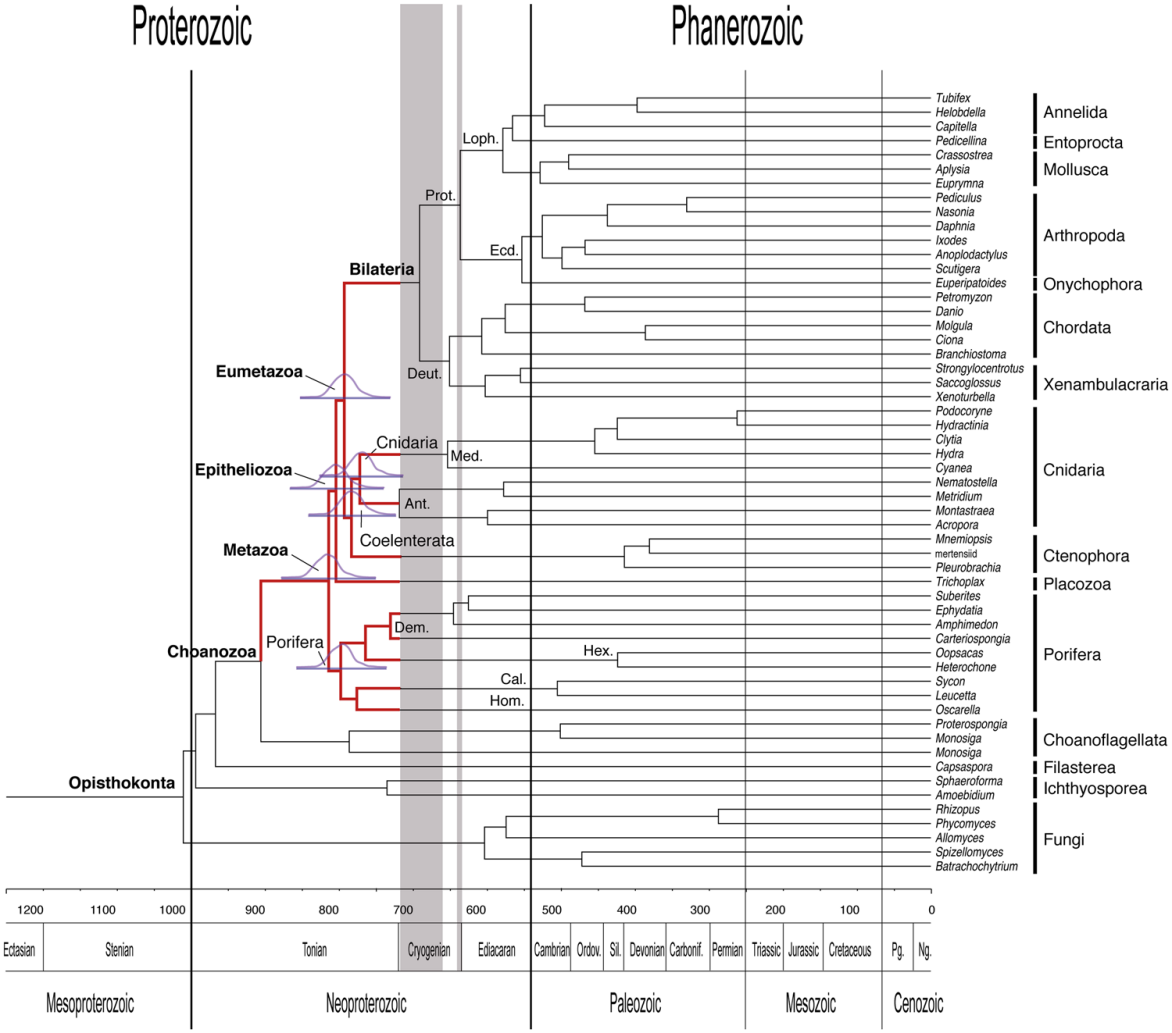
Time-calibrated phylogeny of animals. Phylogeny of crown-Opisthokonta obtained by Philippe *et al*.^18^, time-calibrated using *Calibration set A*, an autocorrelated relaxed clock model, and 1000 Ma root age prior (see text). Gray areas indicate Sturtian (left) and Marinoan (right) glaciations^21^. Thick red branches highlight pre-Snowball Earth radiation of animal lineages. Bars at selected deep nodes represent 95% CrIs; above them density plots highlighting the frequency of different age estimates around the mean are shown (produced with custom python and R scripts developed by S. Vargas). Ages in million years before present (Ma). Stratigraphic abbreviations: Ordov., Ordovician; Sil., Silurian; Carbonif., Carboniferous; Pg., Palaeogene; Ng., Neogene. Taxon abbreviations: Hom., Homoscleromorpha; Cal., Calcarea; Hex., Hexactinellida; Dem., Demospongiae; Ant., Anthozoa; Med., Medusozoa; Deut., Deuterostomia; Prot., Protostomia; Ecd., Ecdysozoa; Loph., Lophotrochozoa.

Changing the phylogenetic position of Ctenophora to sister of the remaining Metazoa or sister to Placozoa + Cnidaria + Bilateria resulted in somewhat different mean age estimates for many nodes (Supplementary Tables S1–S2). However, CrIs for comparable nodes broadly overlapped with those obtained under the topology showing Ctenophora as sister to Cnidaria (=Coelenterata). Importantly, the general pattern of a pre-Sturtian radiation of the main animal lineages was recovered under all three alternative tree topologies (Fig. 4, Supplementary Tables S1–S3, Supplementary Figs S1–S2).

## Discussion

Using Bayesian relaxed molecular-clock dating on a phylogenomic dataset with representative taxon sampling of all five major animal lineages, we have inferred that crown-group animals have a deep pre-Ediacaran origin, which is consistent with several previous studies (reviewed in Sharpe *et al*.^16^; see also dos Reis *et al*.^14^). However, in contrast to these earlier studies, which found a more protracted diversification of non-bilaterian animals, we have inferred a striking pattern of a relatively fast radiation of these lineages and their basic subgroups, prior to the Neoproterozoic Snowball Earth periods (Fig. 4). Although estimates of the exact timing of this radiation differed somewhat depending on model conditions (Figs 1–3), this general result was robust to the choice of molecular clock model, assumptions about the age of the root (=crown-group Opisthokonta), variation in the internal fossil calibrations used, and alternative tree topologies at the base of Metazoa (see also Supplementary Material online for further analyses). Although it is not entirely clear why the pattern reported here has not been found before, it appears likely that insufficient taxon sampling of non-bilaterians in earlier studies allowed only limited conclusions about early animal evolution.

Our estimates are consistent with some palaeontological and geochemical findings interpreted as evidence for Tonian to Cryogenian animal life^31–37^. However, claims of pre-Ediacaran animal remains are still controversial and more work needs to be done to reconcile the fossil and molecular records^7^. Clearly, a more thorough exploration of the Proterozoic fossil record will be necessary to obtain unambiguous evidence for pre-Ediacaran metazoans and a better picture of the morphology and ecology of the early members of the major extant animal lineages. Our results, if accurate, raise important questions such as what triggered this early radiation of animals and how did they survive Snowball Earth? However, future analyses of independent phylogenomic datasets with equally or better suitable taxon sampling, and employing yet other analytical set-ups will be necessary to further test the robustness of these results. Although reconstructing the time-line of animal evolution with high precision might not be possible with current molecular-clock methodology^14^, general patterns such as the one reported here can certainly be detected and will provide a much-needed framework for future research on the origin and early evolution of the Metazoa.

## Electronic supplementary material

SUPPLEMENTARY INFORMATION
