# Supplementary material for: Dating early animal evolution using phylogenomic data
Source: Sci Rep. 2017 Jun 15;7:3599. doi: 10.1038/s41598-017-03791-w (PMC5472626; doi:10.1038/s41598-017-03791-w)
Supplement: Supplementary file 1 — SUPPLEMENTARY INFORMATION [file 41598_2017_3791_MOESM1_ESM.pdf]

SUPPLEMENTARY INFORMATION

Dating early animal evolution using phylogenomic data

Martin Dohrmann and Gert Wörheide

SUPPLEMENTARY METHODS

Details of molecular clock analyses

We used the phylogenomic dataset and the corresponding tree topology (Supplementary Fig. S3) of Philippe et al. [1] as the basis for our divergence-time analyses. We chose this dataset because it was designed to optimize taxon sampling of non-bilaterian animals in order to decipher the phylogeny of the five major animal lineages (Porifera, Placozoa, Ctenophora, Cnidaria, and Bilateria) and was the largest phylogenomic dataset of this kind available when we started to run our analyses. The alignment consists of 30,257 amino acid (aa) positions from 44 metazoan plus 11 non-metazoan opisthokont taxa (see Philippe et al. [1] for details of data acquisition and curation).

Because it has been suggested that ctenophores might not be the sister group of Cnidaria, as in Philippe et al. [1], but sister to Placozoa + Cnidaria + Bilateria [2] or sister to all other animals [3], we also conducted analyses with the tree topology fixed to these alternative arrangements (using TreeEdit [4]) to evaluate the impact of these different tree topologies on divergence time estimation. The analyses with alternative placements of Ctenophora were conducted under the autocorrelated clock model, 1000 Ma root age prior, and *Calibration set A* (see below).

To calibrate the tree (see below for detailed descriptions of the calibration schemes), we initially assembled a set of fossil calibrations from the literature, aiming at maximizing the number of calibrated nodes with a fair distribution across Metazoa, hereafter *Calibration set A*. Because some of the calibrations in *Calibration set A* might be debatable, especially regarding the sponge fossil record, we also assembled a second, more conservative calibration set, *Calibration set B*. Since *Calibration sets A* and *B* contain several maximum constraints for bilaterian nodes (adopted from Rota-Stabelli et al. [5]; see that paper for justification), which might have imposed a strong bias and potentially mislead results, we also conducted an analysis using a modified *Calibration set A* with these constraints removed. This analysis also recovered the general pattern recovered with the other analyses (see Results). However,

overall age estimates for the deepest nodes were roughly 200 myrs older (see output files available at https://doi.org/10.6084/m9.figshare.3472943), which is in our opinion unrealistically ancient. Thus, we did not consider this calibration set further. Finally, we tried to adopt the calibration scheme of Erwin et al. [6] as closely as possible, giving *Calibration set C*.

To assess the sensitivity of the clade age estimates to prior assumptions about the age of the root (i.e., the age of crown-group Opisthokonta), we conducted analyses under three different root age priors (using *Calibration set A*): 1) 1000 ± 100 Ma, adopted from Erwin et al. [6] (see their paper for justification); 2) a somewhat arbitrarily chosen much younger age of 800 ± 100 Ma, which is more in line with interpretations of, e.g., Cavalier-Smith [7]; and 3) 1360 ± 100 Ma, based on molecular-clock estimates for the age of crown-group Opisthokonta [8]. For comparison, we also conducted an analysis using a wider standard deviation (1000 myr), which resulted in only marginally different mean estimates and CrIs (see output files available at https://doi.org/10.6084/m9.figshare.3472943).

To evaluate the relative influence of the calibration priors and the data on the estimated divergence times, we also conducted analyses without data, i.e., sampling only from the prior distributions (using an autocorrelated relaxed-clock model and the 1000 Ma root age prior). For all three calibration sets, the prior mean divergence times and CrIs of the vast majority of internal nodes substantially differed from the posterior estimates (see "prior_comp.txt" files available at https://doi.org/10.6084/m9.figshare.3472943). The only notable exceptions were node 82 (Ambulacraria) in *A* and *B*, node 78 (Chordata) in *B*, and node 69 in *C* (Bivalvia/Gastropoda), all of which are calibrated nodes that are not relevant to our general conclusions. Thus, the calibration sets appeared suitable, allowing the data to dominate the results.

All analyses were performed with PhyloBayes v. 3.3.f [9], because it is the only software that implements the CAT substitution model [10], which was shown to be the best fit for this dataset [1]. We conducted most of the analyses under the lognormal autocorrelated relaxed-clock model (ln), following Lepage et al. [11]. However, because it has been argued that autocorrelated models might not generally provide a good fit to empirical datasets [12,13], we also ran an analysis with *Calibration set A* and the 1000 Ma root age prior under the uncorrelated gamma model (ugam). We further conducted a rough investigation into relative fit of these alternative models to the data, by calculating AICM values [14,15] from the post-burnin likelihood traces using Tracer 1.6 [16]. These analyses suggested strong evidence in favor of the ln model (AICM = 1106); however, this method might be unreliable and less

accurate than more elaborate methods [15,17-19] that are computationally prohibitive for this dataset. Therefore, we present a comparison of results from both above-mentioned analyses, but for the remaining analyses used the ln model because there is at least some evidence for its better fit.

In all analyses, we used the CAT substitution model with a 4-category discrete gamma distribution to account for among-site rate variation [20], and in most analyses we used the default uniform prior for the branching process. We also conducted one analysis (under the 1000 Ma root age prior, the ln clock model, and *Calibration set A*) using the birth-death prior instead, to check for influence of branching process prior choice. This analysis (see output files available at https://doi.org/10.6084/m9.figshare.3472943) suffered from serious convergence problems for certain parameters (although not the likelihood); however, preliminary results show age estimates only slightly different (mostly somewhat younger) than those obtained under the uniform prior for the crucial nodes, and the same general pattern (see Results). Therefore, we did not consider using the birth-death prior further.

To reduce computation time, we invoked the -dc option, i.e., constant (invariable) sites were ignored, resulting in an effective alignment of 20,790 sites. To check if exclusion of constant sites could have biased our results, we also replicated the analysis under *Calibration set A*, the 1000 Ma root age prior, and the ln clock model with constant sites included. This analysis took substantially longer to reach acceptable convergence for all parameters, but it supported the same overall pattern as the analysis excluding constant sites, with only negligible differences in age estimates (see output files available at https://doi.org/10.6084/m9.figshare.3472943). Thus, we are confident that excluding constant sites was also not an issue with the other analyses.

For each analysis, we ran two chains in parallel, sampling every 1000th cycle. Convergence was assessed with the *tracecomp* tool from the PhyloBayes package. We aimed at running the chains until the effective sample size (ESS) of all parameters was >100 and the maxdiff-values had dropped below 0.1, as recommended in the PhyloBayes manual. However, this goal was not achievable in reasonable time for all analyses, so we stopped some chains while some maxdiff-values were still between 0.2 and 0.3 and/or some ESS values between 50 and 100. Posterior estimates of divergence dates were extracted from the chain output using *readdiv* from the PhyloBayes package, using appropriate burn-in determined by plotting parameter values against number of samples. Output files from PhyloBayes, *tracecomp*, and *readdiv* are available in figshare (https://doi.org/10.6084/m9.figshare.3472943).

For interpretation of the chronograms we referred to the 2015 stratigraphic chart of the International Commission on Stratigraphy (ICS 2015); note that this chart differs from the still often used ICS 2014 with respect to the definition of the Tonian/Cryogenian boundary (ICS 2014: 850 Ma, ICS 2015: ~720 Ma) (see http://www.stratigraphy.org/index.php/ics-chart-timescale).

Description of the fossil calibration sets

Note: Taxon names always refer to crown groups and numbers in brackets correspond to node numbers in Supplementary Fig. S3.

Calibration set A.—This set consists of 12 minimum and seven minimum-maximum constraints. For Bilateria, we largely adopted calibrations used by Rota-Stabelli et al. [5] (see that paper for justifications of the minimum and maximum bounds): 581-503 Ma for Ambulacraria (82), 581-519 Ma for Olfactores (Vertebrata + Tunicata; 78), 581-521 Ma for Arthropoda (71), 523-395 Ma for Pancrustacea (72), and 581-532 Ma for Lophotrochozoa (64). In addition, we constrained Annelida (66) at ≥ 518 Ma [21], Hexapoda/Neoptera (73) at ≥ 315 Ma [22], and Vertebrata (79) at ≥ 461 Ma [23]. Among Coelenterata, we assumed ≥ 400 Ma for Ctenophora (92) [24], ≥ 570 Ma for Cnidaria (84) [25], ≥ 500 Ma for Medusozoa (85) [26], ≥ 445 Ma for Hydrozoa (86) [26], ≥ 540 Ma for Anthozoa (89) [27], and ≥ 240 Ma for Scleractinia (91) [23]. Poriferan calibrations included 542-190 Ma for Calcarea (101) [28], ≥ 521 Ma for Homoscleromorpha + Calcarea (100) [29], 542-405 Ma for Hexactinellida (99) [30,31], and ≥ 630 Ma for Silicea *sensu stricto* (95) [32]. For Fungi (105), we used a conservative estimate of ≥ 460 Ma [33]. A more detailed explanation of the individual calibrations is given below.

1. Ambulacraria (82): 581-503 Ma. Adopted from Rota-Stabelli et al. [5].
2. Olfactores (Vertebrata + Tunicata; 78): 581-519 Ma. Adopted from Rota-Stabelli et al. [5].
3. Arthropoda (71): 581-521 Ma. Adopted from Rota-Stabelli et al. [5].
4. Pancrustacea (72): 523-395 Ma. Adopted from Rota-Stabelli et al. [5].
5. Lophotrochozoa (64): 581-532 Ma. Adopted from Rota-Stabelli et al. [5].
6. Annelida (66): ≥ 518 Ma. Based on interpretation of *Phragmochaeta canicularis* from the lower Cambrian (lower to middle Atdabanian) Sirius Passet Lagerstätte (Peary Land, North Greenland) as the oldest known polychaete [21]. Minimum age corresponds to mean age of Cambrian Series 2, Stage 3 (521-514 Ma).

7. Hexapoda/Neoptera (73): ≥ 315 Ma. Based on the oldest known fossils of holometabolan and paraneopteran insects from the Middle Pennsylvanian (Carboniferous, Moscovian) of France [22]. The base of the Moscovian is dated as 315.2 ± 0.2 Ma, hence we used a minimum age of 315 Ma for this node (note that insects branching earlier than crown-group Neoptera were not included by Philippe et al. [1]).
8. Vertebrata (79): ≥ 461 Ma. Based on the oldest known gnathostomes (acanthodians) from the mid-Ordovician (~461 Ma), as suggested in Hedges and Kumar [23].
9. Ctenophora (92): ≥ 400 Ma. Based on interpretation of *Paleoctenophora brasseli* from the Lower Devonian Hunsrück Slate (West Germany) as the oldest known ctenophore with crown-group appearance [24]. The Hunsrück Slate is dated as 408-400 Ma (Late Pragian - Early Emsian), hence we used a minimum age of 400 Ma for crown-Ctenophora.
10. Cnidaria (84): ≥ 570 Ma. Based on interpretation of various microfossils from the Neoproterozoic Weng'an phosphorites (Doushantuo Formation, Southwest China) as crown-group cnidarians [25]. The Doushantuo Formation is dated as 570-580 Ma, hence we used a minimum age of 570 Ma for crown-group Cnidaria.
11. Medusozoa (85): ≥ 500 Ma. The oldest unambiguous crown-group medusozoans are known from the middle Cambrian (Series 3), ~500 Ma [26].
12. Hydrozoa (86): ≥ 445 Ma. The oldest unambiguous crown-group hydrozoans are known from the Upper Ordovician (Hirnantian), ~445 Ma [26].
13. Anthozoa (89): ≥ 540 Ma. Based on *Eolympia pediculata* from the lowest Cambrian (~540 Ma) Kuanchuanpu Formation (Southern China), which was interpreted as a sea anemone (subclass Hexacorallia) by Han et al. [27].
14. Scleractinia (91): ≥ 240 Ma. Based on the appearance of scleractinian reef corals in the Middle Triassic, ~240 Ma, as suggested in Hedges and Kumar [23].
15. Calcarea (101): 542-190 Ma. The minimum constraint is based on *Leucandra walfordi* from the Early Jurassic (~190 Ma) of Northamptonshire (England), which is the oldest known fossil representative of modern calcareous sponges [28]. Although stem-group calcareans might well be older than Cambrian, a Precambrian origin of crown-group Calcarea appears unlikely, so based on similar arguments as applied to Hexactinellida [30], we assigned a maximum constraint of 542 Ma (Ediacaran-Cambrian boundary) to this group.
16. Homoscleromorpha + Calcarea (100): ≥ 521 Ma. According to Xiao et al. [29] the earliest evidence of total-group Calcarea comes from Atdabanian-age strata (Cambrian Series 2, Stage 3), the base of which dates at ~521 Ma. Under the Homoscleromorpha + Calcarea sister-group hypothesis [1,34], the presence of total-group Calcarea implies the presence of total-group

Homoscleromorpha, which justifies this minimum constraint for crown-group Homoscleromorpha + Calcarea.

17. Hexactinellida (99) (= Hexasterophora, since Amphidiscophora was not included by Philippe et al. [1]): 542-405 Ma. For justification of maximum constraint see Dohrmann et al. [30]. The minimum constraint follows from Nose et al. [31], who reported the oldest representative of the order Hexactinosida, *Casearia devonica*, from the Lower Devonian of Northern Spain (Cantabrian Mountains). Although Hexactinosida is paraphyletic [35], dictyonal frameworks like those of *C. devonica* clearly indicate the presence of crown-group Hexasterophora. The fossils were collected from lower-most Emsian strata; since the base of the Emsian is dated 407.6 ± 2.6 Ma, we used a minimum age of 405 Ma for Hexasterophora.

18. Silicea *sensu stricto* (95) (= Hexactinellida + Demospongiae): ≥ 630 Ma. Based on the report of putative triaxonic spicules from the Neoproterozoic Doushantuo Formation (Yangtze Gorges area, South China), which were assigned an age of ~630 Ma [32]. Triaxonic spicules are diagnostic for Hexactinellida; therefore, if the interpretation of Du and Wang [32] is correct, total-group hexactinellids, and therefore crown-group siliceous sponges, must have already existed at that time.

19. Fungi (105): ≥ 460 Ma. Based on interpretation of hyphae and spores from the Middle Ordovician of Wisconsin (~460 Ma) as arbuscular mycorrhizal fungi similar to modern Glomales [33]. To our knowledge the oldest evidence for crown-group Fungi.

Calibration set B.—This calibration set was derived from *Calibration set A* by removing five calibrations that might be considered uncertain or debatable, resulting in seven minimum and five minimum-maximum constraints. The calibrations removed were those for Cnidaria, Anthozoa, as well as all poriferan calibrations. For Silicea *sensu stricto* (= Demospongiae + Hexactinellida), we replaced the ≥ 630 Ma constraint with ≥ 535 Ma, following Antcliffe et al. [36], who argued that hexactinellid sponge spicules of that age from Iran constitute the earliest unambiguous evidence for sponges in the fossil record (see also Muscente et al. [37]).

Calibration set C.—Because of differences in taxon sampling and tree topology, only six (three minimum, two maximum, one minimum-maximum) of the 24 calibrations used by Erwin et al. [6] could be adopted to the phylogeny of Philippe et al. [1]. These were ≤ 713 Ma for Demospongiae (96), ≤ 565 Ma for Ambulacraria (82), ≥ 515 Ma for Arthropoda (71), ≥ 500 Ma for Pancrustacea (72), ≥ 325 Ma for Hexapoda/Neoptera (73), and 548-530 Ma for the Bivalvia/Gastropoda split (69). Note that we performed the analysis with this calibration set

for comparative purposes only; the two maximum constraints used by Erwin et al. are problematic (e.g. Battistuzzi et al. [38]) and these results should therefore be interpreted with caution.

SUPPLEMENTARY TABLES

Supplementary Table S1. Detailed age estimates (in million years ago [Ma]) and associated uncertainty for selected clades of chronogram shown in Fig. S1. stderr, standard error; inf95, lower bound of 95% Credibility Interval (CrI); sup95, upper bound of 95% CrI.

**Clade (crown)**	**mean**	**stderr**	**inf95**	**sup95**
Opisthokonta	1020.41	478.893	936.619	1130.28
Choanozoa	914.127	395.582	849.749	1012.54
Metazoa	850.192	353.248	787.373	937.992
Non-Ctenophora Metazoa	839.659	344.735	777.642	924.856
Placozoa + Cnidaria + Bilateria	829.474	339.881	766.09	912.138
Cnidaria + Bilateria	818.273	326.533	757.604	898.438
Bilateria	684.255	179.296	655.956	728.3
Protostomia	632.06	936.458	614.656	656.586
Deuterostomia	647.588	109.612	627.133	675.9
Cnidaria	783.168	275.057	731.491	847.316
Medusozoa	644.251	19.015	607.694	688.018
Anthozoa	717.023	219.487	679.503	771.423
Porifera	823.266	331.896	761.484	909.316
Silicea s.s.	788.452	308.818	733.523	868.161
Demospongiae	752.227	283.473	703.699	819.708
Hexactinellida	423.561	172.804	405.735	473.374
Calcarea + Homoscleromorpha	800.577	321.948	739.26	882.635
Calcarea	511.767	27.703	432.862	541.594
Ctenophora	432.45	268.883	401.574	514.453

Supplementary Table S2: Detailed age estimates (Ma) and associated uncertainty for selected clades of chronogram shown in Fig. S2. stderr, standard error; inf95, lower bound of 95% CrI; sup95, upper bound of 95% CrI.

Clade (crown)	mean	stderr	inf95	sup95
Opisthokonta	1028.48	528.768	938.811	1150.43
Choanozoa	925.54	443.188	850.314	1033.67
Metazoa	855.059	388.512	789.729	944.879
Epitheliozoa	852.623	386.104	788.188	942.359
Placozoa + Cnidaria + Bilateria	844.578	382.103	780.361	932.353
Cnidaria + Bilateria	834.577	367.338	772.834	920.658
Bilateria	689.25	200.105	656.55	738.787
Protostomia	633.865	104.286	615.577	658.34
Deuterostomia	648.878	125.952	626.96	676.328
Cnidaria	797.056	304.732	744.87	866.884
Medusozoa	651.596	205.476	614.172	696.927
Anthozoa	726.543	233.028	684.571	782.979
Ctenophora	413.576	127.546	400.437	449.249
Porifera	844.545	377.423	782.181	932.515
Silicea s.s.	810.28	346.076	752.002	889.725
Demospongiae	773.109	320.486	720.536	845.087
Hexactinellida	427.087	205.267	405.979	488.817
Calcarea + Homoscleromorpha	823.316	360.951	765.03	905.252
Calcarea	516.772	274.752	441.466	541.344

Supplementary Table S3: Detailed age estimates (Ma) and associated uncertainty for the chronogram shown in Fig. 4. stderr, standard error; inf95, lower bound of 95% CrI; sup95, upper bound of 95% CrI. Node numbers refer to those given in Fig. S3.

**Node**	**mean**	**stderr**	**inf95**	**sup95**
55	1011.12	520.029	921.225	1133.64
56	994.339	492.888	908.527	1121.04
57	967.595	470.691	883.556	1088.4
58	906.14	389.121	836.357	993.024
59	814.483	318.725	757.698	885.34
60	804.635	313.364	747.156	873.861
61	793.404	304.666	738.366	859.852
62	691.422	201.028	658.661	740.049
63	636.468	105.494	618.583	662.357
64	578.82	191.483	574.262	580.932
65	565.827	415.988	556.952	574.276
66	522.334	313.756	518.152	529.975
67	397.234	138.261	362.952	418.649
68	528.462	126.187	493.341	546.094
69	489.98	211.101	435.64	514.944
70	553.125	593.647	543.054	568.397
71	525.497	382.047	521.082	535.921
72	437.503	984.562	418.229	459.094
73	330.162	952.168	316.293	352.709
74	498.913	761.357	484.951	514.695
75	467.683	104.509	447.332	488.168
76	651.025	124.791	629.924	683.949
77	607.404	726.796	590.805	620.494
78	575.763	469.284	563.426	580.876
79	467.986	639.054	461.336	485.921
80	386.061	161.821	350.892	418.487
81	602.76	140.081	571.64	627.536
82	555.032	184.712	511.071	579.718
83	783.699	293.529	730.937	849.112
84	772.305	279.946	721.057	833.529
85	653.764	194.859	619.309	700.584

86	454.574	884.534	445.196	482.574
87	424.114	108.664	405.166	450.562
88	262.135	28.101	190.129	303.195
89	718.859	220.658	679.919	770.219
90	577.729	458.849	475.364	654.642
91	599.659	449.372	496.841	677.36
92	414.518	126.494	400.449	450.316
93	380.588	177.749	347.626	423.721
94	798.208	304.385	743.245	864.822
95	764.712	279.401	715.355	826.308
96	730.883	260.342	681.668	789.532
97	645.615	258.238	595.502	698.616
98	625.492	273.064	569.49	679.097
99	423.633	166.174	405.369	464.983
100	776.331	300.988	719.894	842.821
101	505.107	317.461	434.251	539.995
102	786.62	362.583	716.66	863.299
103	501.073	460.679	398.683	580.287
104	735.537	62.488	607.989	861.422
105	603.58	816.637	475.644	779.783
106	574.575	823.585	442.703	747.969
107	287.381	648.847	130.624	409.806
108	472.016	846.272	332.673	648.935

SUPPLEMENTARY FIGURES

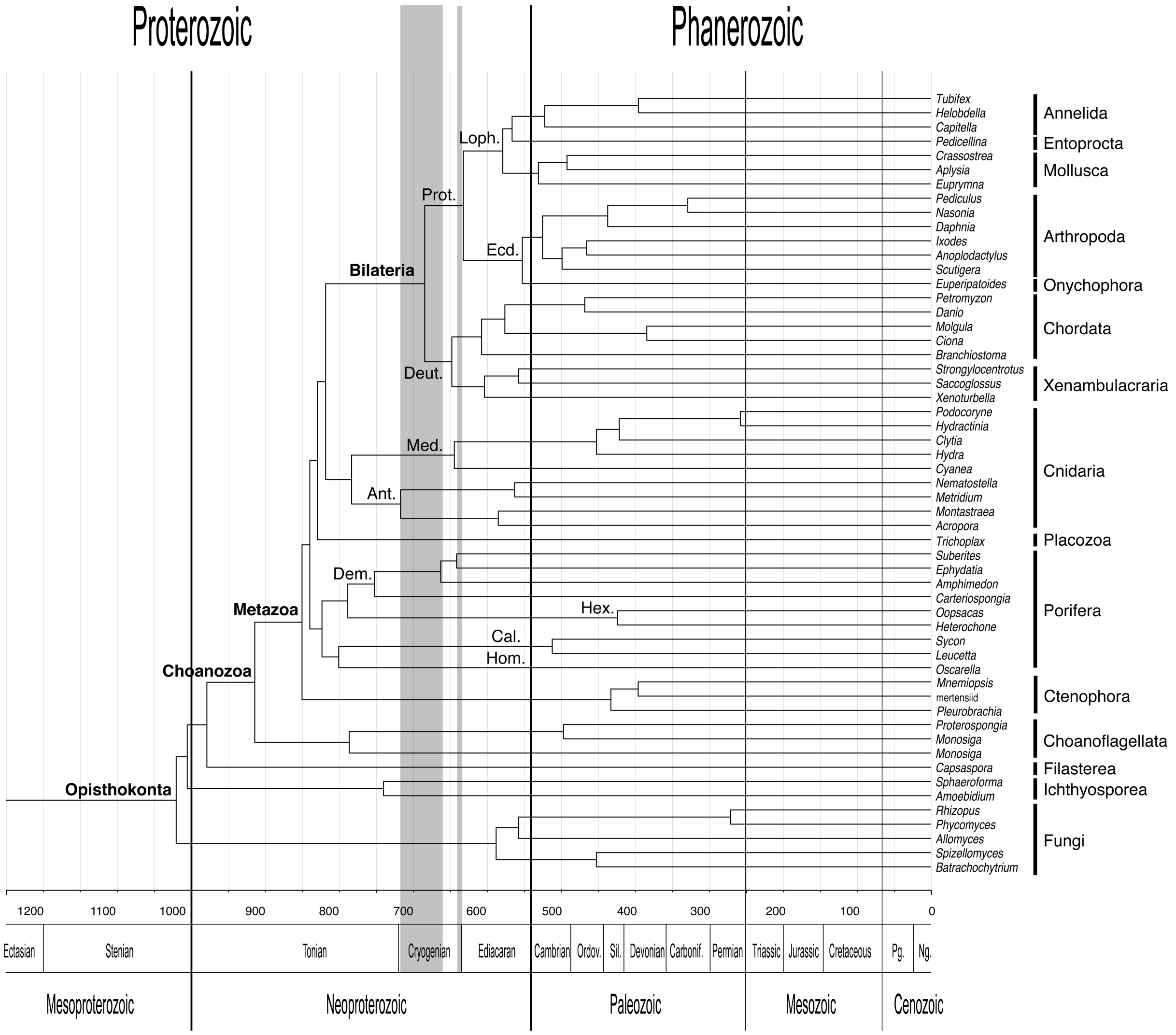

Supplementary Figure S1: Chronogram obtained as in Fig. 4, but with tree topology modified to display Ctenophora as sister to the remaining Metazoa [3]. Gray areas indicate Sturtian (left) and Marinoan (right) glaciations [39]. Ages in million years before present (Ma). Stratigraphic abbreviations: Ordov., Ordovician; Sil., Silurian; Carbonif., Carboniferous; Pg., Paleogene; Ng., Neogene. Taxon abbreviations: Hom., Homoscleromorpha; Cal., Calcarea; Hex., Hexactinellida; Dem., Demospongiae; Ant., Anthozoa; Med., Medusozoa; Deut., Deuterostomia; Prot., Protostomia; Ecd., Ecdysozoa; Loph., Lophotrochozoa.

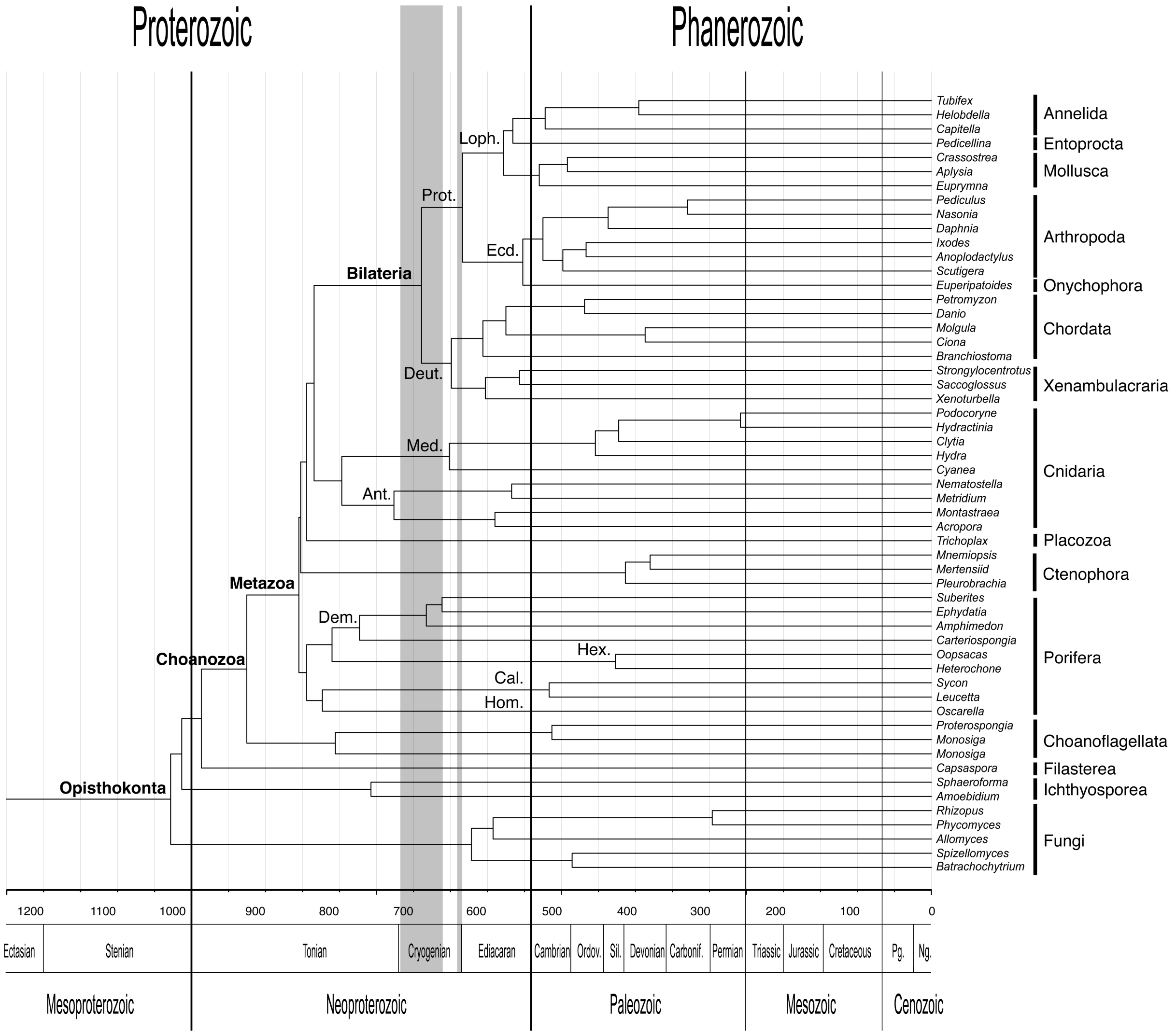

Supplementary Figure S2: Chronogram obtained as in Fig. 4, but with tree topology modified to display Ctenophora as sister to Placozoa + Cnidaria + Bilateria [2]. Gray areas indicate Sturtian (left) and Marinoan (right) glaciations [39]. Ages in million years before present (Ma). Stratigraphic abbreviations: Ordov., Ordovician; Sil., Silurian; Carbonif., Carboniferous; Pg., Paleogene; Ng., Neogene. Taxon abbreviations: Hom., Homoscleromorpha; Cal., Calcarea; Hex., Hexactinellida; Dem., Demospongiae; Ant., Anthozoa; Med., Medusozoa; Deut., Deuterostomia; Prot., Protostomia; Ecd., Ecdysozoa; Loph., Lophotrochozoa.

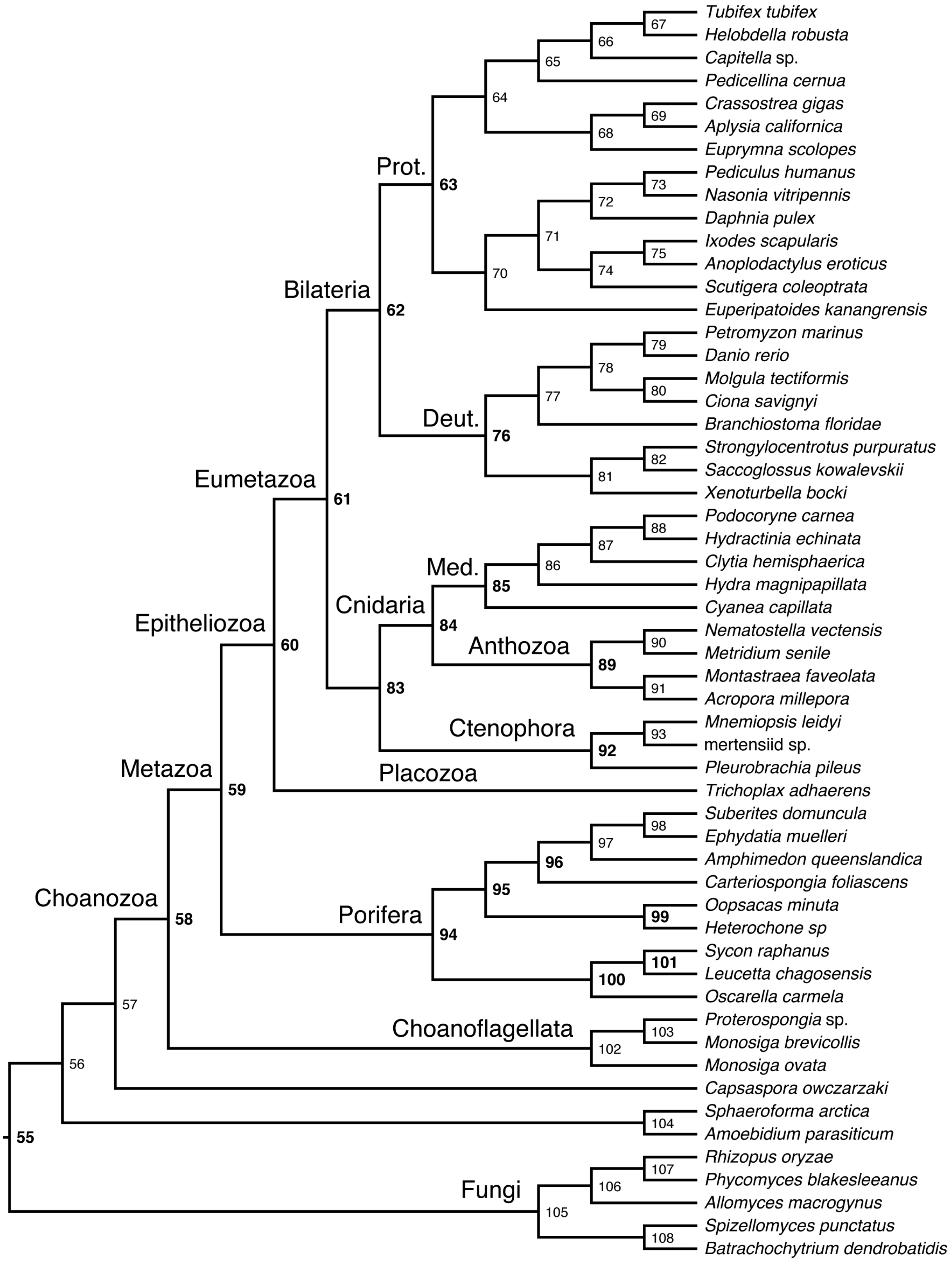

Supplementary Figure S3: Phylogeny of crown-Opisthokonta obtained by Philippe et al. [1], with node numbers referred to in text indicated. Numbers of select nodes shown in Figs. 1-3 displayed in bold. Deut., Deuterostomia; Prot., Protostomia.

SUPPLEMENTARY REFERENCES

1. Philippe, H. et al. Phylogenomics revives traditional views on deep animal relationships. *Curr. Biol.* **19**, 706-712 (2009).
2. Pisani, D. et al. Genomic data do not support comb jellies as the sister group to all other animals. *Proc. Natl. Acad. Sci. USA* **112**, 15402-15407 (2015).
3. Borowiec, M. L., Lee, E. K., Chiu, J. C. & Plachetzki, D. C. Extracting phylogenetic signal and accounting for bias in whole-genome data sets supports the Ctenophora as sister to remaining Metazoa. *BMC Genomics* **16**, 987 (2015).
4. Rambaut, A. & Charleston, M. TreeEdit. *Available at http://evolve.zoo.ox.ac.uk/software/TreeEdit/main.html* (2002).
5. Rota-Stabelli, O., Daley, A. C. & Pisani, D. Molecular timetrees reveal a Cambrian colonization of land and a new scenario for ecdysozoan evolution. *Curr. Biol.* **23**, 392-398 (2013).
6. Erwin, D. H. et al. The Cambrian conundrum: early divergence and later ecological success in the early history of animals. *Science* **334**, 1091-1097 (2011).
7. Cavalier-Smith, T. Deep phylogeny, ancestral groups and the four ages of life. *Phil. Trans. R. Soc. B* **365**, 111-132 (2010).
8. Parfrey, L. W., Lahr, D. J. G., Knoll, A. H. & Katz, L. A. Estimating the timing of early eukaryotic diversification with multigene molecular clocks. *Proc. Natl. Acad. Sci. USA* **108**, 13624-13629 (2011).
9. Lartillot, N., Lepage, T. & Blanquart, S. PhyloBayes 3: a Bayesian software package for phylogenetic reconstruction and molecular dating. *Bioinformatics* **25**, 2286-2288 (2009).
10. Lartillot, N. & Philippe, H. A Bayesian mixture model for across-site heterogeneities in the amino-acid replacement process. *Mol. Biol. Evol.* **21**, 1095-1109 (2004).
11. Lepage, T., Bryant, D., Philippe, H. & Lartillot, N. A general comparison of relaxed molecular clock models. *Mol. Biol. Evol.* **24**, 2669-2680 (2007).
12. Ho, S. Y. W. An examination of phylogenetic models of substitution rate variation among lineages. *Biol. Lett.* **5**, 421-424 (2009).
13. Linder, M., Britton, T. & Sennblad, B. Evaluation of Bayesian models of substitution rate evolution—parental guidance versus mutual independence. *Syst. Biol.* **60**, 329-342 (2011).
14. Raftery, A., Newton, M., Satagopan, J. & Krivitsky, P. in *Bayesian Statistics* (eds Bernardo, J. M., Bayarri, M. J. & Berger, J. O.) 1-45 (Oxford University Press, Oxford, 2007).
15. Baele, G. et al. Improving the accuracy of demographic and molecular clock model comparison while accommodating phylogenetic uncertainty. *Mol. Biol. Evol.* **29**, 2157-2167 (2012).
16. Rambaut, A., Suchard, M. & Drummond, A. J. Tracer v1.6. *Available from http://tree.bio.ed.ac.uk/software/tracer/* (2013).
17. Lartillot, N. & Philippe, H. Computing Bayes factors using thermodynamic integration. *Syst. Biol.* **55**, 195-207 (2006).
18. Xie, W., Lewis, P. O., Fan, Y., Kuo, L. & Chen, M.-H. Improving marginal likelihood estimation for Bayesian phylogenetic model selection. *Syst. Biol.* **60**, 150-160 (2011).
19. Baele, G., Li, W. L. S., Drummond, A. J., Suchard, M. A. & Lemey, P. Accurate model selection of relaxed molecular clocks in Bayesian phylogenetics. *Mol. Biol. Evol.* **30**, 239-243 (2013).
20. Yang, Z. Maximum likelihood phylogenetic estimation from DNA sequences with variable rates over sites: approximate methods. *J. Mol. Evol.* **39**, 306-314 (1994).

21. Conway Morris, S. & Peel, J. S. The earliest annelids: Lower Cambrian polychaetes from the Sirius Passet Lagerstätte, Peary Land, North Greenland. *Acta Palaeontol. Pol.* **53**, 137-148 (2008).
22. Nel, A. et al. The earliest known holometabolous insects. *Nature* **503**, 257-261 (2013).
23. Hedges, S. B. & Kumar, S. *The Timetree of Life* (Oxford University Press, Oxford, 2009).
24. Stanley Jr., G. D. & Stürmer, W. The first fossil ctenophore from the Lower Devonian of West Germany. *Nature* **303**, 518-520 (1983).
25. Chen, J.-Y. et al. Precambrian animal life: probable developmental and adult cnidarian forms from Southwest China. *Dev. Biol.* **248**, 182-196 (2002).
26. Young, G. A. & Hagadorn, J. W. The fossil record of cnidarian medusae. *Palaeoworld* **19**, 212-221 (2010).
27. Han, J. et al. Tiny sea anemone from the lower Cambrian of China. *PLoS One* **5**, e13276 (2010).
28. Pickett, J. in *Systema Porifera. A Guide to the Classification of sponges* (eds Hooper, J. N. A. & van Soest, R. W. M.) 1117-1119 (Plenum, New York, 2002).
29. Xiao, S., Hu, J., Yuan, X., Parsley, R. L. & Cao, R. Articulated sponges from the Lower Cambrian Hetang Formation in southern Anhui, South China: their age and implications for the early evolution of sponges. *Palaeogeogr. Palaeocl. Palaeoecol.* **220**, 89-117 (2005).
30. Dohrmann, M., Vargas, S., Janussen, D., Collins, A. G. & Wörheide, G. Molecular paleobiology of early-branching animals: integrating DNA and fossils elucidates the evolutionary history of hexactinellid sponges. *Paleobiology* **39**, 95-108 (2013).
31. Nose, M., Vodrazka, R., Fernández, L.-P. & Méndez-Bedia, I. First record of chambered hexactinellid sponges from the Palaeozoic. *Acta Palaeontol. Pol.* **59**, 985-996 (2014).
32. Du, W. & Wang, X. L. Hexactinellid sponge spicules in Neoproterozoic dolostone from South China. *Paleont. Res.* **16**, 199-207 (2012).
33. Redecker, D., Kodner, R. & Graham, L. E. Glomalean fungi from the Ordovician. *Science* **289**, 1920-1921 (2000).
34. Dohrmann, M., Janussen, D., Reitner, J., Collins, A. G. & Wörheide, G. Phylogeny and evolution of glass sponges (Porifera, Hexactinellida). *Syst. Biol.* **57**, 388-405 (2008).
35. Dohrmann, M. et al. An integrative systematic framework helps to reconstruct skeletal evolution of glass sponges (Porifera, Hexactinellida). *Frontiers Zool.* **14**, 18 (2017).
36. Antcliffe, J. B., Callow, R. H. T. & Brasier, M. D. Giving the early fossil record of sponges a squeeze. *Biol. Rev.* **89**, 972-1004 (2014).
37. Muscente, A. D., Michel, F. M., Dale, J. G. & Xiao, S. Assessing the veracity of Precambrian 'sponge' fossils using *in situ* nanoscale analytical techniques. *Precamb. Res.* **263**, 142-156 (2015).
38. Battistuzzi, F. U., Billing-Ross, P., Murillo, O., Filipski, A. & Kumar, S. A protocol for diagnosing the effect of calibration priors on posterior time estimates: A case study for the Cambrian explosion of animal phyla. *Mol. Biol. Evol.* **32**, 1907-1912 (2015).
39. Rooney, A. D., Strauss, J. V., Brandon, A. D. & Macdonald, F. A. A Cryogenian chronology: Two long-lasting synchronous Neoproterozoic glaciations. *Geology* **43**, 459-462 (2015).
